# Effect of the Velvet Antler of Formosan Sambar Deer (*Cervus unicolor swinhoei*) on the Prevention of an Allergic Airway Response in Mice

**DOI:** 10.1155/2012/481318

**Published:** 2012-12-23

**Authors:** Ching-Yun Kuo, Ting Wang, Ting-Yeu Dai, Chih-Hua Wang, Kun-Nan Chen, Yen-Po Chen, Ming-Ju Chen

**Affiliations:** ^1^Taiwan Livestock Research Institute, Council of Agriculture, Tainan 71246, Taiwan; ^2^Department of Animal Science and Technology, National Taiwan University, No. 50, Lane 155, Section 3 Keelung Road,Taipei 10617, Taiwan; ^3^Department of Mechanical Engineering, Tungnan University, Taipei 22202, Taiwan; ^4^Center for Biotechnology, National Taiwan University, No. 81, Changxing, Taipei 10617, Taiwan

## Abstract

Two mouse models were used to assay the antiallergic effects of the velvet antler (VA) of Formosan sambar deer (*Cervus unicolor swinhoei*) in this study. The results using the ovalbumin- (OVA-) sensitized mouse model showed that the levels of total IgE and OVA-specific IgE were reduced after VA powder was administrated for 4 weeks. In addition, the *ex vivo* results indicated that the secretion of T helper cell 1 (Th1), regulatory T (Treg), and Th17 cytokines by splenocytes was significantly increased (*P* < 0.05) when VA powder was administered to the mice. Furthermore, OVA-allergic asthma mice that have been orally administrated with VA powder showed a strong inhibition of Th2 cytokine and proinflammatory cytokine production in bronchoalveolar fluid compared to control mice. An increase in the regulatory T-cell population of splenocytes in the allergic asthma mice after oral administration of VA was also observed. All the features of the asthmatic phenotype, including airway inflammation and the development of airway hyperresponsiveness, were reduced by treatment with VA. These findings support the hypothesis that oral feeding of VA may be an effective way of alleviating asthmatic symptoms in humans.

## 1. Introduction

Velvet antler (VA), the unossified antler from members of the family Cervidae, has been used in traditional Chinese medicines and health foods for over 2000 years [1]. VA has been examined for various effects, including anti-inflammatory effects [2], antioxidant properties, antilipid peroxidation properties [3], and anti-infective effects [1]. Many reports and clinical observations have convincingly shown that VA and its extracts are able to alleviate the symptoms of rheumatoid arthritis [4, 5], osteoarthritis, and osteoporosis [6, 7], are able to promote dermal cell proliferation and angiogenesis [8, 9], and can be used to treat heart failure [10]. Although the detailed mechanisms of the health benefits exerted by VA have not yet been clarified, the immunoregulation effects induced by VA, such as the inhibition of the proinflammatory cytokines and T helper 1 (Th1) cells in serum and in arthritic joints [1, 5], the upregulation of monocytes, and the enhancement of the phagocytic activity [11, 12], are considered to play key roles in the health benefits.

The possible bioactive components of VA that contribute to these activities have been investigated. The phosphates and gangliosides of VA are able to ease the symptoms of senility [13, 14]. Various polysaccharides and lysophosphatidylcholines have been shown to be responsible for antiulcer and antihypertensive activity, respectively [15]. Phosphatidylcholines with saturated fatty acyl chains seem to act as immunostimulating factors [16]. Other components of VA, such as polypeptides [17], sterols, and inorganic substances (Ca, Zn, and Pb) [18, 19], may be involved in other health benefits. These include, recently, a novel 3.2 kDa polypeptide from VA of red deer (*Cervus elaphus* Linnaeus) that is able to stimulate the growth of rat epidermal cells and rabbit costal chondrocytes in a dose-dependent manner [17].

Formosan sambar deer (*Cervus unicolor swinhoei*) is an indigenous subspecies of deer found in Taiwan [20]. The use of its VAs as an immune booster and as an antiallergy agent has steadily increased since the start of deer farming in Taiwan in 1963; however, few studies have examined the efficacy of VA supplementation with respect to the prevention and treatment of allergic disorders. Allergic asthma, a type 1 hypersensitivity, is characterized by an allergen-induced chronic inflammation of the lungs together with airway hyperresponsiveness (AHR); it is also associated with an enhancement of allergen-induced eosinophilia, goblet cell hyperplasia, increased allergen-specific immunoglobulin (Ig) E levels, increased T helper cell 2 (Th2) dominant cytokine production, and lymphocyte infiltration into the airways. In our previous study, we demonstrated the protective mechanisms that are induced by the VA in a *Staphylococcus aureus *infected animal model [1]. The protective mechanisms of VA included inducing the production of various T regulatory cytokines [1]. This feature might also be related to the antiallergic effects of VA. Thus we have further investigated in this study the effects of VA of Formosan sambar deer (*Cervus unicolor swinhoei*) on the prevention of allergy and asthma.

## 2. Materials and Methods

### 2.1. VA Samples

VA from Formosan sambar deer was harvested in Kaohsiung Animal Propagation Station, Taiwan Live Stock Research Institute (Pintong, Taiwan) after a 75-day VA growing period. Fresh VA was immediately sliced and frozen at −20°C. The frozen VA slices were dehydrated by lyophilization (Freeze dryer, Kingmech Co. Ltd., Taipei, Taiwan) and then ground into a fine powder (VA powder) by a pulverizing machine using a less than 100 mesh (No. RT-02A, Rong Tsong Co., Taichung, Taiwan).

For the *in vitro* assays, the dehydrated VA extract was dissolved in Dulbecco's phosphate-buffered saline (DPBS; Thermo Fisher Scientific Inc., Logan, UT, USA) and sterilized by passing through a 0.22 *μ*m filter (Millipore Corp., Carrigtwohill, Ireland). For the experiments performed here, the extract sample was dissolved and diluted immediately before the assays were performed.

### 2.2. Experimental Animals

Six-week-old female BALB/cByJNarl mice (National Laboratory Animal Center; NLAC, Taipei, Taiwan) were accustomed to their new environment for at least 1 week before the start of the experiment. The mice were maintained in an automatic light/dark cycle (light periods of 14 h). Temperature and humidity were kept constant at 22°C and 50%, respectively. The animal care and treatment were performed in accordance with the guidelines of the National Science Council of Republic of China.

### 2.3. OVA-Sensitized Mouse Model

An experimental allergic mouse model, as described by Pichavant et al. [21] and Hong et al. [22], was used in this study with minor modifications. The mice were intragastrically (IG) fed with 2.5, 5, or 10 mg of VA powder dissolved in 200 *μ*L PBS daily throughout the experimental period. The mice fed with 200 *μ*L distilled water with or without OVA sensitization were used as positive and negative control groups, respectively. Sera were collected on days 0, 14, 21, and 28 by submandibular bleeding in order to allow total and OVA-specific IgE quantification. On day 28, the mice were sacrificed and their splenocytes harvested for *ex vivo* cytokine analysis.

### 2.4. Allergic Asthma Mouse Model

The ovalbumin- (OVA-) sensitized mouse model of allergic airway inflammation was used in this study and has been described in detail by Lee et al. [23] and Hong et al. [24]. The mice were fed with 10 mg of VA powder/mouse/day throughout the experimental period. Initially, the mice were sensitized by intraperitoneal injection of 20 *μ*g OVA (Sigma, Grade V). The protocol was similar to the OVA-sensitized mouse model. On days 28, 29, and 30, the mice were challenged with OVA (1% in PBS) or PBS by ultrasonic nebulization (Aerogen Ireland Ltd., Galway, Ireland) for 20 min per mouse. On day 32, the airway responsiveness of the mice was measured. Blood was then collected by submandibular bleeding before the mice were sacrificed.

### 2.5. Airway Responsiveness

At 24 hours after the final OVA challenge, *in-vivo* lung function measurement was performed by whole-body plethysmography (Buxco WBP, Buxco. Electronics, Wilmington, NC, USA) as described by Karimi et al. [25]. The slope of the dose response was calculated by linear regression between the measured airway resistance and the log10-transformed methacholine dose (12.5, 25, and 50 mg/mL; Sigma).

### 2.6. Preparation of Splenocytes

The mice were sacrificed by cervical dislocation, and splenocytes were harvested for culture. The preparation of the splenocyte cultures followed the procedure described by Hong et al. [24]. Briefly, The spleens were removed and placed in 10 mL of RPMI-1640 medium (HyClone, Logan, UT, USA) supplemented with 1% antibiotics (50 *μ*g/mL penicillin, 50 *μ*g/mL streptomycin sulfate, and 100 *μ*g/mL neomycin sulfate; Invitrogen, Carlsbad, CA, USA). The spleens were cut into pieces then pressed through a 70 *μ*m cell strainer (BD Falcon, Bedford, MA, USA). The cells were rinsed with 10 mL RPMI-1640 medium with 1% antibiotic then centrifuged (800 ×g) at room temperature for 5 min. The cell pellet was collected and resuspended in 5 mL RBC lysis buffer for 2 minutes. After washing and centrifugation, the cell pellet was resuspended in 5 mL RPMI-1640 medium with 1% antibiotic and 10% heat-inactivated fetal bovine serum (FBS, Invitrogen, Carlsbad, CA, USA) and adjusted to 2 × 10^5^ cells/mL in 24-well culture plate.

 The isolated splenocytes from different treatment groups were treated with ovalbumin (100 *μ*g/mL) for 48 hours. After treatment, the cell meda were collected and centrifugated at 10,000 ×g for 3 min to discard cells. The supernatants were stored in −80°C freezer.

### 2.7. Bronchoalveolar Lavage (BAL) and Lung Histology

After sacrificing the treated mice, their lungs were rinsed with 1 mL PBS. Bronchoalveolar fluid was obtained by aspirating three times via tracheal cannulation. The cells in the BAL fluid were removed by centrifugation at 200 ×g for 15 min, and then the samples were resuspended in PBS (1 mL). The supernatants were stored at −80°C until their cytokine content could be measured [26]. After removing the BAL, the lungs of the mice were inflated with 10% formalin (Wako, Chuo-Ku, Osaka, Japan), fixed for 24 hours, and embedded in paraffin. The fixed and embedded tissue was then stained with hematoxylin and eosin (Sigma) to allow histological assessment using light microscopy (Optima, Aurora, CO, USA). Periodic acid-Schiff (PAS) stain (IMEB Inc., San Marcos, CA, USA) was also applied in order to measure goblet cell and mucus production.

### 2.8. Measurement of the Levels of Cytokines and Ova-Specific IgE

The levels of various cytokines (IL-4, IL-5, IL-13, IL-12p40, IL-6, IL-1*β*, tumor necrosis factor-*α* (TNF-*α*), and IFN-*γ*) were assessed using the R&D system (Minneapolis, MN, USA). The results are expressed as the concentration of each cytokine (pg/mL). Blood samples from the treated mice were collected by submandibular bleeding, coagulated for 1 h at room temperature and then subsequently centrifuged for 5 min at 17,500 ×g. OVA-specific IgE was determined using an OVA-specific IgE kit (Serotec, Oxford, United Kingdom). 

### 2.9. Statistical Analysis

All results were analyzed using the general linear model procedure available from the Statistical Analysis System software package version 8.1. Duncan's multiple range test was used to detect differences between the treatment means. Each experiment was conducted in triplicate.

## 3. Results

### 3.1. VA Powder of Formosan Sambar Deer Reduced the IgE and Spleen Weight of Mice Using the OVA-Sensitized Model

**Figure 1:**
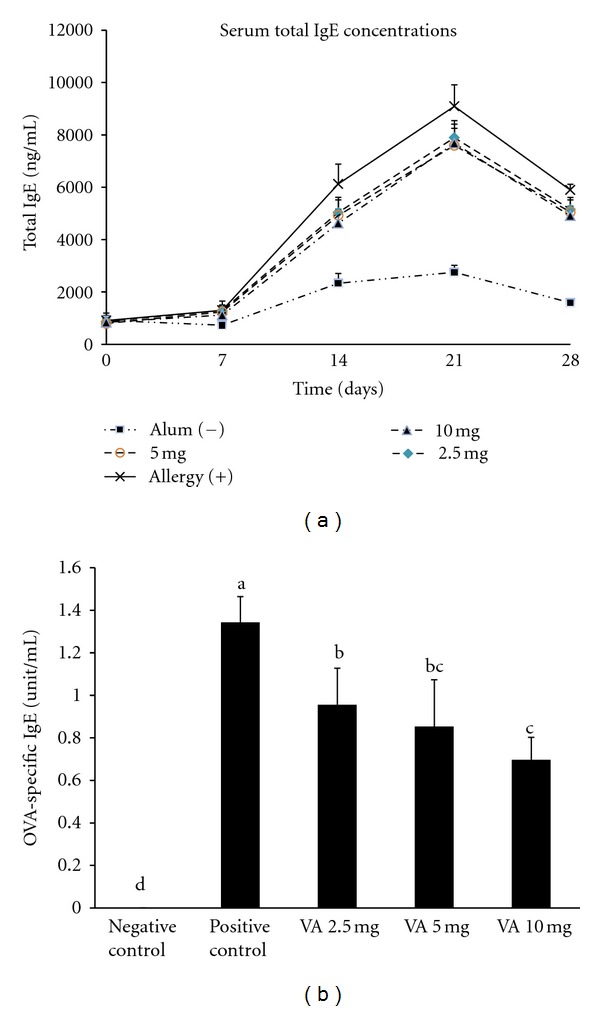
Serum total immunoglobulin E (IgE) concentrations (a) and ovalbumin- (OVA-) specific IgE titer (b) in serum of OVA-sensitized mice-administrated different dosages of the velvet antler (VA) powder. Values are mean + SEM (a–c) Means without the same superscripts differ significantly (*P* < 0.05).

To assess the effect of VA powder in terms of protection against an allergic response, the level of total serum IgE antibodies was assessed every week in this study (Figure 1(a)). The titer of total serum IgE was elevated after the 1st sensitization. Treatment with VA powder was able to significantly inhibit this increase in serum total IgE on day 14, 21, and 28. The level of OVA-specific IgE on day 28 was also significantly suppressed (*P* < 0.05) in the serum of OVA-sensitized mice treated with VA (Figure 1(b)). A higher dosage apparently resulted in a greater IgE inhibition effect. The spleen weight of OVA sensitized mice was examined after sacrifice (data not shown). The spleen weight of the mice was significantly increased (*P* < 0.05) after OVA sensitization. However, when sensitized mice were treated with VA powder, there was a significant decrease in spleen weight (*P* < 0.05). On the other hand, the body weight changes were not significantly different among all treatment groups (data not shown).

### 3.2. VA from Formosan Sambar Deer Affects the Production of the Cytokines Th1, Th2, Treg, and Th17 by the Splenocytes of Mice Using the OVA-Sensitized Model

**Figure 2:**
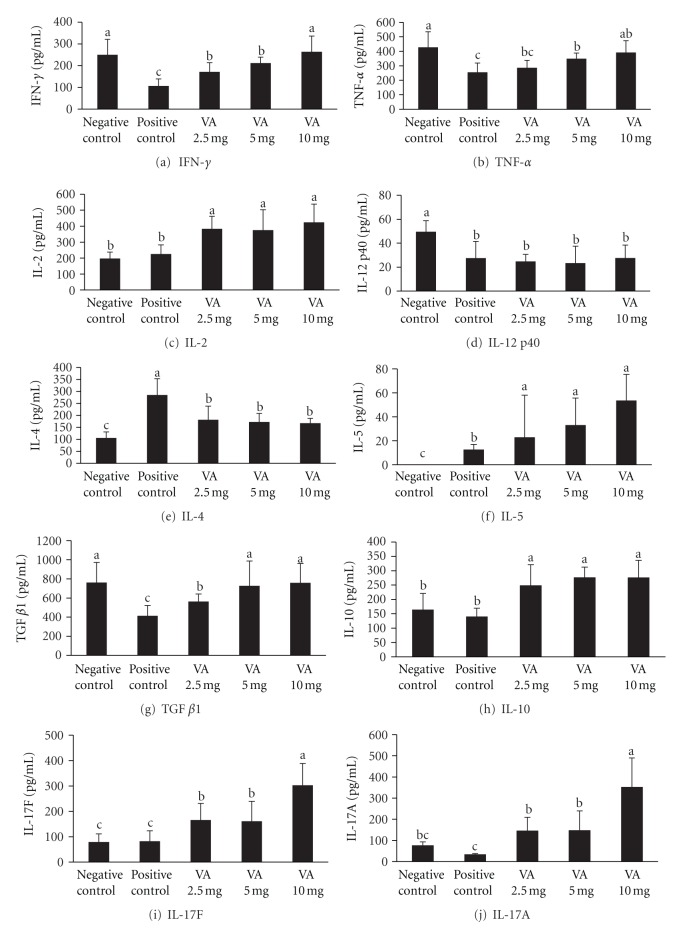
*Ex vivo* secretion of T helper (Th)-1 cytokines of splenocytes of ovalbumin- (OVA-) sensitized mice-administrated different dosages of velvet antler (VA) powder. Values are mean + SEM (a–c) Means without the same superscripts differ significantly (*P* < 0.05), *n* = 8.

To clarify the mechanisms involved in the inhibition of IgE production in OVA-sensitized mice, we examined the levels of the cytokines Th1, Th2, Treg, and Th17 in splenocytes from OVA-sensitized mice *ex vivo*. When the Th1 cytokines were examined (Figures 2(a)–2(c)), the levels of IFN-*γ*, TNF-*α*, and IL-2 in the splenocytes from mice fed with VA powder were significantly higher (*P* < 0.05) compared to the OVA positive-control mice, and this increase occurred in a dose-responsive manner. However, there was no significant difference in IL-12 level between the positive control group and the VA powder group (Figure 2(d)). In contrast to the above findings, production of the Th2 cytokine, IL-4, was inhibited in splenocytes when OVA-sensitized mice were fed with VA powder (Figure 2(e)). However, production of another Th2 cytokine, IL-5, showed no difference between the two groups (Figure 2(f)).

When the Treg cytokines were examined (Figures 2(g) and 2(h)), the secretion of IL-10 and TGF-*β*1 by splenocytes was significantly attenuated (*P* < 0.05) by treatment with the VA powder, and this occurred in a dose-responsive manner. Similar effects were also observed for the Th17 cytokines (Figures 2(i) and 2(j)) with significantly higher levels (*P* < 0.05) of IL-17A and IL-17F being observed in splenocytes of the VA-treated group when compared with the OVA positive-control group. These findings demonstrate that oral administration of the VA powder results in an antiallergic effect on the OVA-sensitized mice model, and that this effect occurs in a dose-responsive manner. To further evaluate the possibility of the VA being able to protect against an allergic-airway response, we selected a 10 mg/day/mouse model to explore the effect of VA on the level of IgE antibodies in serum, on the level of IgE antibodies in BAL, on lung histology, and on the airway response.

### 3.3. VA of Formosan Sambar Deer Reduced IgE Production in Serum and Attenuated Th1, Th2, and Th17 Levels in the Splenocytes Using an Allergic Asthma Mouse Model

**Figure 3:**
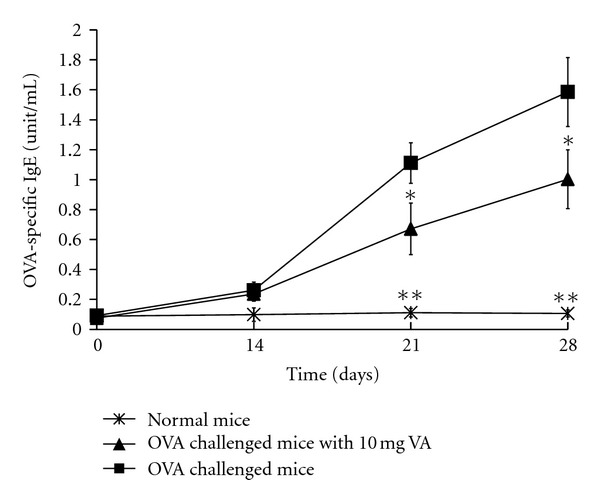
Effect of 10 mg velvet antler (VA) powder on ovalbumin- (OVA-) specific immunoglobulin E (IgE) productions in serum of OVA-sensitized mice after OVA challenge. Asterisk denotes values that are significantly different from positive control (OVA sensitized and challenged, *P < 0.05, **P < 0.01).

**Figure 4:**
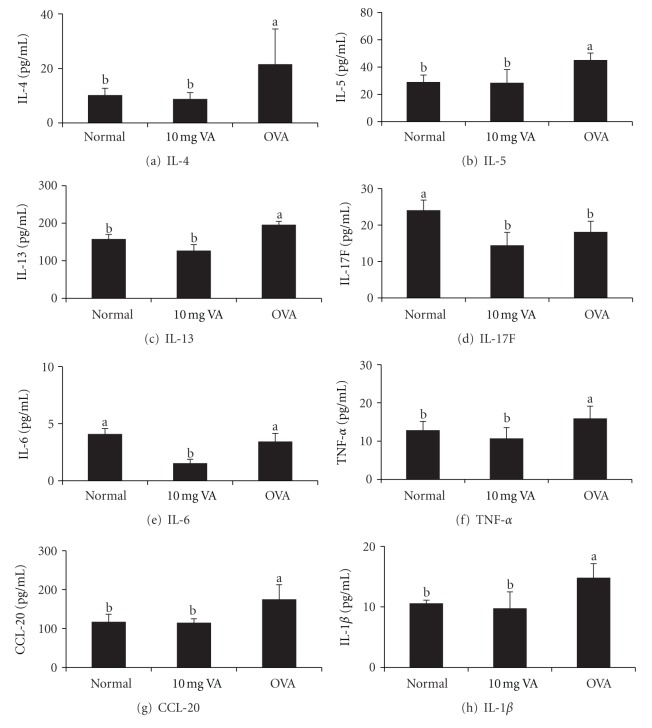
Effect of oral administration of 10 mg velvet antler (VA) powder on T helper (Th) 2, Th17, and proinflammatory cytokine secretion in bronchoalveolar lavage of ovalbumin-(OVA-) sensitized mice after OVA challenge. Values are mean + SEM (a–c) Means without the same superscripts differ significantly (*P* < 0.05), *n* = 8.

Mice that were orally fed with 10 mg of VA powder showed a significant reduction in OVA-specific IgE levels (*P* < 0.01) on day 21 and 28 (Figure 3) when compared with the OVA-allergic asthma mice. To assess whether treatment with VA powder was able to protect against an allergic airway response, Th2 and Th1 cytokine levels were also determined for splenocytes from the OVA-allergic asthma mice* ex vivo*. The results showed an increase in splenocyte production of various Th2 cytokines (IL-4, IL-5, and IL-13) and various Th1 cytokines (IL-1*β*, IL-6, and TNF-*α*), while Th17 cytokines were found to be significantly attenuated in splenocytes (*P* < 0.01) of the OVA-allergic asthma mice pretreated with 10 mg/day/mouse of VA powder (Figure 4).

### 3.4. VA of Formosan Sambar Deer Attenuated the Airway Response and Allergic Lung Symptoms Using an Allergic Asthma Mouse Model

**Figure 5:**
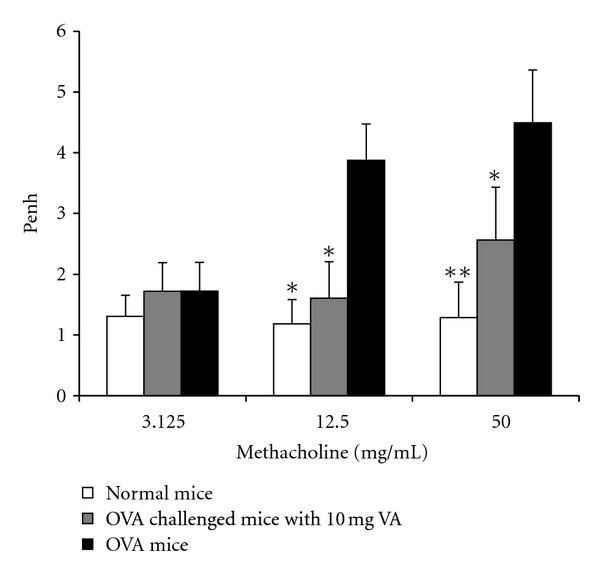
Effect of oral administration of 10 mg velvet antler (VA) powder on the airway response to aerosolized methacholine measured 24 h after the last ovalbumin (OVA) challenge in OVA-sensitized mice as expressed by Penh. The positive controls (PC) were fed with 200 *μ*L of phosphate buffer saline (PBS) and sensitized and challenged with OVA. The negative controls (NC) were fed with 200 *μ*L of PBS and sensitized and challenged with PBS. Values are means ± SEM of 8 to 10 mice per group. *Significant difference from positive control (*P* < 0.01).

The airway response showed that the OVA-allergic asthma mice had a significant increase in enhanced pause (Penh) in response to doses of aerosolized methacholine compared with the normal negative controls (*P* < 0.01). The development of this increased airway response was significantly attenuated when the mice were pretreated with 10 mg/day/mouse of the VA powder (Figure 5).

**Figure 6:**
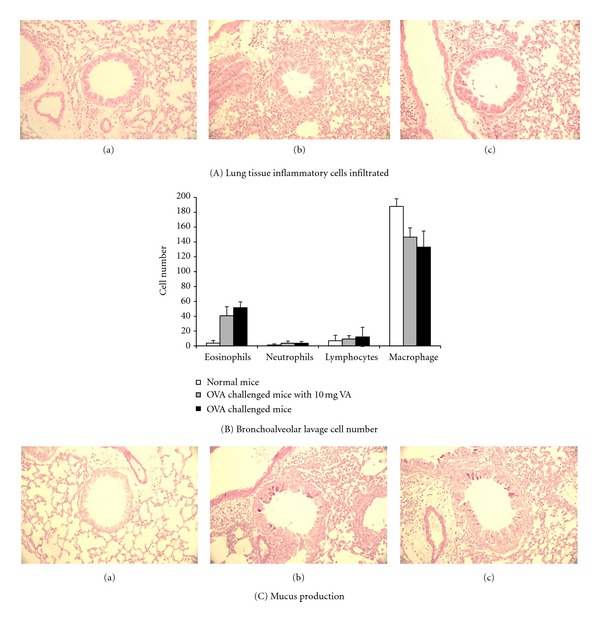
Effect of oral administration of 10 mg velvet antler (VA) powder on (A) inflammatory cells infiltration in lung tissue and (B) bronchoalveolar lavage cell number, and (C) mucus production in ovalbumin- (OVA-) sensitized mice after OVA challenge. Representative sections of lung tissue from (a) negative control, (b) positive control, and (c) oral administration of 10 mg VA powder.

The histological results indicated that OVA aerosol challenge induced marked infiltration of inflammatory cells into the peribronchiolar and perivascular connective tissue (Figure 6(A)(a)). The majority of the infiltrated inflammatory cells were monocytes and eosinophils. The increase in eosinophils in the lung parenchyma of the OVA-allergic asthma mice was significantly reduced by treatment with 10 mg/day/mouse of VA powder (Figure 6(A)(b)). These results were confirmed by the bronchoalveolar lavage cell numbers (Figure 6(B)). Eosinophil numbers were significantly decreased when OVA-allergic asthma mice were pretreated with 10 mg/day/mouse of VA powder.

PAS staining was used to evaluate goblet cell hyperplasia, and it was found that there was an overproduction of mucus together with the presence of goblet cell hyperplasia in the bronchial airways of OVA-allergic asthma mice and that this was absent in normal mice (Figures 6(C)(a) and 6(C)(b)). The mice that had been orally treated with VA powder showed a significant suppression of the increase in mucus production and goblet cell hyperplasia (Figure 6(C)(c)).

## 4. Discussion

In the present study, we used two mouse models to assay the effects of VA of Formosan sambar deer (*Cervus unicolor swinhoei*) with respect to preventing allergic and asthma effects. The OVA-sensitized model clearly demonstrated that orally administered VA powder seemed to have an antiallergic effect via a suppression of OVA-specific IgE production (Figure 1) and the prevention of an increase in spleen weight. Serum IgE is one of the most important clinical markers for allergic responses. Such a reduction in serum OVA-specific IgE is likely to relieve the allergic syndrome mediated by mast cells [27]. An increase in spleen weights reflects the inflammatory condition of the spleen [28]. Normally, allergen-specific naïve and memory T cells in the various lymph nodes, including the spleen, are stimulated to proliferate after exposure to an allergen. The above findings suggest that the proliferation of T cells is being prevented by the oral administration of VA.

The proliferation of T cells should also trigger a Th2 type inflammatory response, predominately involving IL-4 and IL-5 [29], which then skews the Th1/Th2 balance toward Th2 responses. The Th1/Th2 balance is important in allergic diseases [27]. The present findings indicated that the oral administration of 5 mg or more of VA powder is able to elevate the levels of Th1, Treg, and Th17 cytokines in splenocytes, which results in a inhibition of the production of Th2 cytokines in the OVA-sensitized mice (Figure 2). These changes shift the Th1/Th2 response toward a more balanced state. Among the Th1 cytokines that were increased, it is known that IFN-*γ* is an essential cytokine for the development of Th1-mediated cell immunity, which helps to eliminate intracellular pathogens via the activation of macrophages and an increase in phagocytosis as well as higher production of IL-12, NO, and superoxide [30, 31]. In addition to the above, the changes would further favor the development of Th1 cells themselves and repress the development of Th2 cells [31]. The secretion of another Th1 cytokine, IL-2, was also significantly elevated in the VA powder-treated mice (Figure 2(c)). IL-2 is an autocrine growth factor for T cells and promotes the survival, growth, and differentiation of T cells [32].

The anti-inflammatory regulatory cytokines, IL-10 and TGF-*β*, which are produced by regulatory T cells [33], have been reported to be be able to alleviate allergic inflammation [34]. IL-10 is a key regulator of allergic diseases, not only because it downregulates the Th2 cell-derived cytokines IL-4 and IL-5 [35], but also because it directly inhibits the IgE-induced degranulation of mast cells, which leads to a reduced secretion of the inflammatory mediator histamine [36]. Furthermore, TGF-*β*, a potent regulatory cytokine, is able to initiate the differentiation of Treg and Th17 cells, repress the proliferation of B lymphocyte progenitors, and inhibit the activation of T cells and macrophages [37, 38]. Th17 cells are a relatively newly defined Th cell subset. The role of Th17 cells and Th17 cytokines in allergic diseases is still unclear [39]; however, some studies have indicated that Th17 cells may be involved in inhibiting the production of the Th2 cytokines, IL-4 and IL-5 [40, 41].

Taking the above findings together, it would seem that oral administration of VA powder for 28 days is able to regulate the Th1/Th2 balance by augmentation of the Th1, Th17, and Treg cytokines in splenocytes in a dose-dependent manner when an OVA-sensitized model is used. Therefore, we further assessed the anti-allergic asthma effects of 10 mg of VA powder using the well-established OVA-induced asthma model. Allergic asthma is characterized by AHR to a variety of allergens, chronic pulmonary eosinophilia, an elevated serum IgE, and excessive airway mucus production [42]. Oral administration of VA powder was found to lead to a suppression of all the above features of the asthmatic phenotype, including specific IgE production, airway inflammation, and the development of AHR (Figures 5 and 6).

The suppression of all the symptoms of asthma in the present study was associated with reduced levels of various Th1 cytokines (TNF-*α*, IL-6, and IL-1*β*), with reduced levels of various Th2 cytokines (IL-4, IL-5 and IL13) and with reduced levels of a Th17 cytokines (IL-17F) in splenocytes (Figure 4). The effects on the Th1 and Th17 cytokines in OVA-induced asthma model did not parallel exactly the results of OVA-sensitized mouse model due to the different mechanisms of both models. Since the OVA-sensitized mouse model induces whole body allergy at an early stage, augmentation of the Th1 and Th17 cytokines in splenocytes may skew the Th1/Th2 balance toward a Th1 response to prevent the allergic symptoms. On the other hand, the increased levels of Th1 and Th17 cytokines in OVA-induced asthma model seem to act on epithelial cells and attenuate the excessive host inflammatory responses associated with allergic asthma [43]. Previous studies have also observed an increased release of TNF-*α* [44], IL-1*β* [45] and IL-6 [46] in BAL of asthmatic patients. Laan et al. [47] reported that free soluble IL-17 is produced during the severe inflammation that is characterized by high neutrophil numbers in human airways.

The regulatory effects of the VA powder on immunity have been reported in other studies. Dai et al. [1] found that the protective mechanisms induced by VA in *S. aureus*-infected animals might include dual roles as an immune enhancer and as a proinflammatory cytokine suppressor at the later stages of bacterial infection. Kim et al. [5] found that an IP injection of VA extract was able to prevent the development of arthritis in rats and suppress proinflammatory cytokine secretion by lymph node cells. In another study, the proliferation of resting splenocytes was significantly increased when they were treated with VA extract [48]. Furthermore, Suh et al. [49] showed that VA seemed to act as an immune enhancer and improve phagocytosis. Many water-insoluble substances found in VA, such as gangliosides, a sialic acid-containing glycosphingolipid [50], chondroitin sulfate, and fatty acids, have been suggested as acting to modulate the immune response. VA also contains a range of other water soluble bioactive substances, such as carbohydrates, hexosamines, hydronyprolines, mucopolysaccharide, sialic acids, and uronic acids, and, some of which may be involved in the modulation of immune systems. However, the specific bioactive compounds that are related to VA's anti-allergic effects still need to be further investigated.

## 5. Conclusion 

When taken together, the results presented here clearly demonstrate that oral administration of VA powder is able to prevent and reduce the OVA-induced allergic symptoms and AHR in mice. The prevention by VA powder of allergen-induced sensitization occurs via an elevation of Treg, Th1, and Th17 activity, via a suppression of Th2 cytokines in splenocytes and via an inhibition of specific IgE production in serum. In contrast, the alleviation of airway disease was found to be related to a suppression of Th2, proinflammatory, and Th17 cytokines in splenocytes and to an inhibition of specific IgE production in serum; these changes lead to a reduction in the AHR to methacholine and a reduction in lung inflammation in OVA-allergic asthma mice. Thus, there is strong support for the hypothesis that the oral intake of VA powder from Formosan sambar deer is likely to be an effective approach to the prevention of allergy and the alleviation of asthmatic symptoms. To the best of our knowledge, this is the first paper that completely explores the antiallergic asthma effects of VA powder.
